# Surgical training rotation design: effects of hospital type, rotation theme and duration

**DOI:** 10.1002/bjs5.50326

**Published:** 2020-07-24

**Authors:** D. B. T. Robinson, L. Hopkins, O. P. James, C. Brown, A. G. M. T. Powell, S. Hemington‐Gorse, T. Abdelrahman, W. G. Lewis, R. J. Egan

**Affiliations:** ^1^ Wales Postgraduate Medical and Dental Education School of Surgery Health Education and Improvement Wales Nantgarw UK; ^2^ Department of Surgery, Morriston Hospital Swansea UK; ^3^ Swansea Medical School, Swansea University Swansea UK; ^4^ Division of Cancer and Genetics Cardiff University, Heath Park Cardiff UK

## Abstract

**Background:**

Entrants into UK surgical specialty training undertake a 2‐year programme of core surgical training, rotating through specialties for varying lengths of time, at different hospitals, to gain breadth of experience. This study aimed to assess whether these variables influenced core surgical trainee (CST) work productivity.

**Methods:**

Intercollegiate Surgical Curriculum Programme portfolios of consecutive CSTs between 2016 and 2019 were examined. Primary outcome measures were workplace‐based assessment (WBA) completion, operative experience and academic outputs (presentations to learned societies, publications and audits).

**Results:**

A total of 344 rotations by 111 CSTs were included. Incremental increases in attainment were observed related to the duration of core surgical training rotation. The median number of consultant‐validated WBAs completed during core surgical training were 48 (range 0–189), 54 (10–120) and 75 (6–94) during rotations consisting of 4‐, 6‐ and 12‐month posts respectively (*P* < 0·001). Corresponding median operative caseloads (as primary surgeon) were 84 (range 3–357), 110 (44–394) and 134 (56–366) (*P* < 0·001) and presentations to learned societies 0 (0–12), 0 (0–14) and 1 (0–5) (*P* = 0·012) respectively. Hospital type and specialty training theme were unrelated to workplace productivity. Multivariable analysis identified length of hospital rotation as the only factor independently associated with total WBA count (*P* = 0·001), completion of audit (*P* = 0·015) and delivery of presentation (*P* = 0·001) targets.

**Conclusion:**

Longer rotations with a single educational supervisor, in one training centre, are associated with better workplace productivity. Consideration should be given to this when reconfiguring training programmes within the arena of workforce planning.

## Graphical abstract

This study aimed to assess whether hospital status, rurality, duration of hospital rotations and specialty theme of core surgical training had an impact on workplace productivity.

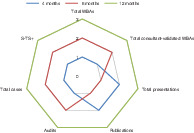

Longer placements improve outcomes

## Abstract (es)

**Antecedentes:**

Los residentes de especialidades quirúrgicas del Reino Unido realizan un período troncal de formación quirúrgica de 2 años, en el que rotan por diversas especialidades durante periodos de tiempo variables y en diversos hospitales, a fin de conseguir una experiencia amplia. Este estudio tuvo como objetivo evaluar si estas variables influyeron en la productividad de los residentes durante el período troncal (*core surgical trainee*, CST).

**Métodos:**

Se examinaron los inventarios de los programas del *Intercollegiate Surgical Curriculum Programme* (ISCP) de CST consecutivos entre 2016 y 2019. Las variables principales fueron la puntuación final del *Workplace‐Based Assessment* (WBA), y la actividad quirúrgica y académica (presentaciones a sociedades académicas, publicaciones y auditorías) realizadas.

**Resultados:**

Se incluyeron 344 rotaciones de 111 CST. Se constataron mejores resultados en relación con la duración de la rotación de CST. La mediana (rango) de la puntuación de los supervisores en las WBA fue de 48 (0’189), 54 (10’120) y 75 (6’94) (*P* < 0,001) en las rotaciones a los 4, 6 y 12 meses, respectivamente. El número de intervenciones (como cirujano principal) fue de 84 (3’357), 110 (44’394) y 134 (56’366) (*P* < 0,001) y de presentaciones a sociedades científicas fue de 0 (0‐12), 0 (0‐ 14) y 1 (0‐5) (*P* = 0,012). No hubo relación entre el tipo de hospital o la especialidad y la productividad en el lugar de trabajo. El análisis multivariable identificó la duración de la rotación como único factor independientemente relacionado con la puntuación de la WBA (*P* = 0,001), la finalización de la auditoría (*P* = 0,015) y el número de presentaciones realizadas (*P* = 0,001).

**Conclusión:**

Las rotaciones de periodos de tiempo largos con un solo supervisor y en un solo centro se asocian con una mejor productividad en el lugar de trabajo. Debería tenerse en cuenta este factor al reconfigurar los programas de capacitación desde el punto de vista laboral.

## Introduction

Seldom has surgical training been under such scrutiny or faced such radical change in the UK as at present. The Shape of Training and Improving Surgical Training (IST) pilot programmes both aim to deliver improvements in the quality of training, a better balance between service and training for trainees, and professionalization of the role of the surgical trainer. Yet no consensus exists regarding surgical rotation design and, in particular, the duration of individual hospital placements. Currently, entrants into UK surgical specialty training have first to complete a core surgical training programme prior to application and progression to higher surgical training. During this challenging 2‐year period, trainees typically rotate through different specialties for varying lengths of time, at different locations, to gain a breadth of experience^1^. In Wales, trainees typically rotate through three specialties within the 2‐year programme, with 12–16 months spent within their chosen specialty theme. Despite this prolonged, focused time in the individual's chosen specialty, myriad variables can influence the training quality and opportunities within different hospitals, in varied settings, and often with training overseen by multiple educational supervisors. This can lead to lack of continuity, a disjointed experience and is likely, at least in part, to be responsible for the low satisfaction levels identified by the General Medical Council's annual national training survey, which reported that core surgical trainees are among the least satisfied, with overall satisfaction rates of 77·2 per cent compared with anaesthesia (85·6 per cent) and general practice (88·6 per cent)^2^.

Abdelrahman and colleagues^3^ in 2016 demonstrated that success in being appointed to higher surgical training was associated with greater operative experience, demonstrated by fuller logbooks, more workplace‐based assessments (WBAs)^4^ and academic productivity^3^. The aim of this study was to assess whether the experiences of trainees in terms of hospital type and location, specialty theme and duration of rotations impacted on completion of WBAs, academic productivity and operative experience.

## Methods

Rotations undertaken between 2016 and 2019 in a single UK deanery were analysed. Data regarding workplace productivity were collected using Head of School function within the Intercollegiate Surgical Curriculum Programme (ISCP) profiles of the trainees. These profiles are used in the UK to record trainees' progression and academic achievements.

Workplace productivity was assessed in three broad categories: number of WBAs (total and consultant‐validated) with subgroup analysis of case‐based discussions (CBD), clinical evaluation exercises (CEX), and direct observation of procedural skills (DOPS)/procedural‐based assessments (PBA); operative experience consisting of total cases with separate analysis of operations in which the trainee was the primary operating surgeon with or without supervision (supervised – trainer scrubbed+ (S‐TS+)); and academic productivity, comprising audits, number of presentations to learned societies and peer‐reviewed publications.

For each of these variables, comparisons were made between teaching hospitals (associated with a university) and rural and urban district general hospitals (DGHs), specialty theme variation, and the varying lengths of rotations. Specialty themes included general surgery, trauma and orthopaedics, ear, nose and throat, urology, maxillofacial surgery, plastic surgery and neurosurgery. The impact of undertaking rotations across multiple sites in a single year of training was also assessed. Formal permission under the ISCP data governance structure was not required because the study represented service evaluation.

### Defining rurality

The Office for National Statistics uses a number of measures of rurality, which include settlement size (fewer than 10 000 persons), sparsity measures, land measures, dispersion measures, access measures, or a combination of these^5^, ^6^. Referring to these definitions, rural hospitals were defined as a DGH requiring a significant commute from a major city, serving a largely rural population; not all DGHs therefore fulfilled the criteria for rurality, a method described previously^7^. One DGH in the study had the same bed capacity as one of the university teaching hospitals, yet was potentially busier with regard to emergency surgical admissions. The concept of rurality was therefore used to correct for potential confounding factors during data analysis.

### Equating outcome measures in varying lengths of rotations

Rotation length varied between rotations (4, 6 or 12 months). To facilitate comparison and limit bias, all data were equated to a 12‐month rotation period by dividing the outcome measures by the duration in months of the associated rotation, and applying a multiplication factor of 12. A radar plot was created, producing a visual representation of rankings with regard to the productivity variables studied over the varying durations of rotations; each duration of rotation was given a score based on the median for each parameter studied (3, highest score; 2, middle score; 1, lowest score).

### Markers of satisfactory progression

The Joint Committee on Surgical Training (JCST) outlines the minimum requirements for operative caseload experience in a single year (at least 120 cases). However, definitive numbers of WBAs, audits, presentations to learned societies and peer‐reviewed publications required for successful progression to the next year of training are not provided. A simple statement declares that there is a requirement for ‘evidence of engagement’ with each of these components^8^. Health Education and Improvement Wales School of Surgery defines targets for trainees to ensure satisfactory progress, which include a minimum of 60 total and 30 consultant‐validated WBAs, and delivery of at least one audit and one presentation per academic year. The above deanery productivity targets, coupled with the JCST operative experience expectations, were used to facilitate multivariable analysis of the factors associated with success in each of these domains. No targets were set for peer‐reviewed publications, although encouraged, and therefore a figure of at least one publication was used as a benchmark figure for analytical purposes.

### Statistical analysis

Shapiro–Wilk analysis was used to assess normality in each data cohort, and statistical analysis was performed in SPSS® version 25 (IBM, Armonk, New York, USA). Data are presented, unless indicated otherwise, as median (range) values. Mann–Whitney *U* and Kruskal–Wallis tests were used to perform univariable analysis of factors associated with each category compared. Multivariable analyses were performed using binary logistic regression to identify variables independently associated with greater achievement in the productivity fields studied. Variables with a *P* < 0·100 following univariable analysis were included in the multivariable analysis, using a forward conditional model.

## Results

Some 344 rotations undertaken by 111 consecutive core surgical trainees (CSTs) were included in the study.

The median number of overall CBD + CEX, consultant‐validated CBD + CEX, overall DOPS + PBA, consultant‐validated DOPS + PBA, total WBAs and total consultant‐validated WBAs were 38 (range 0–120), 30 (0–105), 30 (0–150), 21 (0–117), 69 (0–261) and 51 (0–189) respectively. Median total and S‐TS+ operative caseload experience was 252 (54–737) and 87 (3–394) respectively. The median number of audits, presentations to learned societies, and peer‐reviewed publications were 0 (0–9), 0 (0–14) and 0 (0–6) respectively.

### Outcomes related to duration of rotation placements

**Fig. 1:**
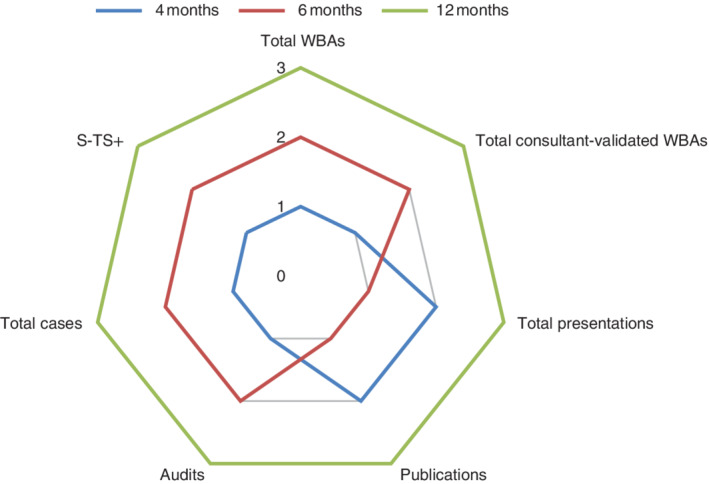
Duration of rotation as a comparator for academic achievements in core surgical training Radar chart comparing duration of rotations with regard to workplace productivity. WBA, workplace‐based assessments; S‐TS+, supervised – trainer scrubbed or greater (all adjusted to provide 12‐month equivalent outputs).

*Table* *1* outlines the variations in WBA completion, operative experience and academic productivity related to the duration of rotations. *Fig*. *1* provides a radar plot of rotation duration, identifying the incrementally higher levels of achievement associated with longer rotational placements.

**Table 1:** Comparison of workplace‐based assessments, operative experience and academic output related to duration of rotation

	Duration of rotation			
	4 months (*n* = 285)	6 months (*n* = 37)	12 months (*n* = 22)	*P* *
CBD + CEX	36 (0–120)	42 (6–92)	49 (4–87)	0·002
Consultant‐validated CBD + CEX	27 (0–105)	32 (6–74)	48 (4–58)	< 0·001
DOPS + PBA	30 (0–150)	32 (4–82)	30 (3–66)	0·945
Consultant‐validated DOPS + PBA	21 (0–117)	24 (4–58)	27 (2–36)	0·049
Total WBAs	69 (0–261)	74 (10–158)	80 (7–138)	0·131
Total consultant‐validated WBAs	48 (0–189)	54 (10–120)	75 (6–94)	< 0·001
Total operative cases	249 (54–582)	252 (134–492)	271 (144–737)	0.264
S‐TS+ operative cases	84 (3–357)	110 (44–394)	134 (56–366)	< 0·001
Audit	0 (0–9)	0 (0–6)	1 (0–3)	0·328
Presentations	0 (0–12)	0 (0–14)	1 (0–5)	0·012
Publications	0 (0–6)	0 (0–2)	0 (0–2)	0·232

Values are median (range). CBD, case‐based discussion; CEX, clinical evaluation exercises; DOPS, direct observation of procedural skills; PBA, procedure‐based assessment; WBA, workplace‐based assessment; S‐TS+, supervised – trainer scrubbed or greater.

* Kruskal–Wallis test.

### Relative performance of different hospital types

No significant differences were observed in any of the domains of WBAs, operative experience or academic productivity when comparing teaching hospitals with DGHs (*Table* *2*). Similar findings were seen when comparing rural and urban hospitals with respect to WBAs, with the exception of total CBD + CEX completion. Moreover, CSTs in urban DGHs performed significantly more operations as the primary surgeon, despite equivalent overall operative caseloads (*Table* *3*).

**Table 2:** Comparison of workplace‐based assessments, operative experience and academic output related to hospital status

	Teaching hospital (*n* = 175)	District general hospital (*n* = 169)	*P* *
CBD + CEX	36 (3–87)	39 (0–120)	0·112
Consultant‐validated CBD + CEX	27 (0–74)	30 (0–105)	0·151
DOPS + PBA	30 (0–150)	30 (0–141)	0·865
Consultant‐validated DOPS + PBA	21 (0–117)	21 (0–90)	0·311
Total WBAs	69 (7–225)	72 (0–261)	0·232
Total consultant‐validated WBAs	51 (6–177)	54 (0–189)	0·089
Total operative cases	261 (54–737)	248 (63–501)	0·075
S‐TS+ operative cases	96 (3–366)	81 (3–394)	0·232
Audit	0 (0–9)	0 (0–9)	0·967
Presentations	0 (0–14)	0 (0–12)	0·586
Publications	0 (0–6)	0 (0–3)	0·681

Values are median (range). CBD, case‐based discussion; CEX, clinical evaluation exercises; DOPS, direct observation of procedural skills; PBA, procedure‐based assessment; WBA, workplace‐based assessment; S‐TS+, supervised – trainer scrubbed or greater.

* Mann–Whitney *U* test.

**Table 3:** Comparison of workplace‐based assessments, operative experience and academic output related to rurality of district general hospital

	Urban (*n* = 296)	Rural (*n* = 48)	*P* *
CBD + CEX	39 (0–120)	29 (3–99)	0·050
Consultant‐validated CBD + CEX	30 (0–105)	24 (3–99)	0·092
DOPS + PBA	30 (0–150)	28 (3–117)	0·556
Consultant‐validated DOPS + PBA	21 (0–117)	21 (0–90)	0·318
Total WBAs	72 (0–261)	68 (6–201)	0·188
Total consultant‐validated WBAs	51 (0–189)	53 (6–144)	0·874
Total operative cases	252 (54–737)	240 (96–477)	0·366
S‐TS+ operative cases	92 (3–394)	66 (3–285)	0·001
Audit	0 (0–9)	0 (0–6)	0·102
Presentations	0 (0–14)	0 (0–6)	0·153
Publications	0 (0–6)	0 (0–3)	0·703

Values are median (range). CBD, case‐based discussion; CEX, clinical evaluation exercises; DOPS, direct observation of procedural skills; PBA, procedure‐based assessment; WBA, workplace‐based assessment; S‐TS+, supervised – trainer scrubbed or greater.

* Mann–Whitney *U* test.

### Influence of training theme

No significant differences were seen in any of the domains of WBAs or academic productivity related to specialty‐themed programmes (*Table* *S1*, supporting information). There was, however, significant variance regarding primary surgeon operative experience, with oral and maxillofacial surgery‐themed trainees gaining the most experience and neurosurgery‐themed trainees the least.

### Influence of multiple hospital rotations

Forty‐one of the 111 CSTs (36·9 per cent, 117 rotations) undertook training across multiple training sites in a single academic year. Comparisons were therefore made with rotations confined to a single hospital site, and no variations in productivity were observed in this regard.

### Multivariable analysis

Six separate multivariable analyses were performed against the workplace productivity criteria for success at the annual review of competency progression (WBA at least 60, consultant‐validated WBA at least 30, total operative caseload of at least 120, at least 1 audit, at least 1 presentation to learned societies, and at least 1 publication). Only length of hospital rotations was independently associated with total WBA of 60 or more (odds ratio (OR) 5·38, 95 per cent c.i. 1·91 to 15·13; *P* = 0·001), one or more audits (OR 1·63, 1·10 to 2·43; *P* = 0·015) and one or more presentations (OR 2·02, 1·34 to 3·00; *P* = 0·001). No factors were independently associated with consultant‐validated WBA of 30 or more, total operative caseload of 120 or more, or one or more publications.

## Discussion

The principal finding of this study assessing the impact of training rotation variables on workplace productivity in the medical arena was the finding of substantial productivity variation in relation to the duration of training placements. Significant incremental differences were identified between 4‐, 6‐ and 12‐month rotations. Specifically, total consultant‐validated WBA numbers varied by 27 procedures (56·3 per cent; *P* < 0·001), total operative experience by 22 cases (8·8 per cent) and primary surgeon operative experience by 50 cases (60 per cent; *P* < 0·001). For all key outcomes measured, a 1‐year placement outperformed both a 4‐ and a 6‐month rotation, although hospital status was not associated with differential attainment. Moreover, in multivariable analysis, duration of hospital rotations was the only factor independently associated with a total WBA number of 60 or more (*P* = 0·001), at least one audit (*P* = 0·015) and at least one presentation to a learned society (*P* = 0·001).

The variation in durations of surgical rotations is not unique to UK surgical training. In the USA and Canada, postgraduate residency programmes comprise 5 years of rotations that typically range from 4 to 26 weeks. Furthermore, and in contrast to the UK where minimal variation is seen between different deanery programmes, in North American systems the employing hospital has greater governance over the structure of postgraduate surgical training, with the common goal of supplying opportunities to meet a standardized curriculum^9^, ^10^, ^11^, ^12^, ^13^, ^14^. Australia has a similar system to that in the UK, with rotations typically lasting 6–12 months in one unit before moving to another hospital, in order to gain a breadth of experience in different environments. As such, the finding of greater productivity from longer rotations in a single unit with a single educational supervisor could have global importance^15^.

There is a paucity of objective evidence in the current literature supporting the educational benefit of longer placements for training within the postgraduate medical arena. An editorial^16^ in the *British Medical Journal* suggested a number of solutions to improve recruitment and retention of trainees, including reducing the frequency of rotation within contemporary training schemes and a move to reinstate a more stable team‐based working environment. Historically, the perceived negative effect of regular rotations was mitigated by the benefit of the old ‘firm’ structure, in which daily patient management and on‐call commitments were the responsibility of the same team. This structure has now disappeared, but the frequent rotations remain.

In the undergraduate arena, longitudinal integrated clerkships have been a hot topic of discussion for some time. Although differing in structure from contemporary surgical training placements, the underlying positive impacts of having the same educational/clinical supervisors for a prolonged period of time, often 9–12 months, echo true in the uplift in output measures demonstrated in the present study. Ellaway and co‐workers^17^ highlighted the benefit of longer clerkships in allowing continuity of supervision and development of a better teacher–student relationship. This message was further emphasized by Hirsh *et al*.^18^, who stated that longer placements provide students with the emotional support to take intellectual risks to enhance their learning, while the development of trusting relationships and common goals promotes a culture of coaching, effective feedback and enhanced clinical performance. An opinion piece^19^ in *The New England Journal of Medicine* strongly supported these arguments, as well as further emphasizing the lack of published literature in this area. A study by Teherani and colleagues^20^ found that teachers appreciated the opportunity to see their assigned student's development over time and felt they had greater influence on a student's learning during longer rotations. It has also been identified that, in these prolonged relationships, trainers feel more able to modify their training style to suit the student's needs better; this should facilitate improved learning and professional development^21^. The present study provides objective quantitative evidence to support these qualitative findings.

The latest iteration of surgical training in the UK has seen the widespread adoption of the pilot IST programme, the surgical arm of the Shape of Training review. IST promotes longer rotations to develop the relationship between the trainer and trainee to one of ‘mentorship, coaching and supervision’. It also highlights that there is still debate on how long is ideal, but suggests a minimum of 12 months in a single institution^2^. Although pilot IST data have not yet been released, the data presented in the present study will no doubt reinforce the concept of longer durations to ensure continuity within a stable training environment.

A common counterargument against longer rotations/infrequent movement is that trainees may not gain the same breadth of experience^17^, ^22^, ^23^. This study addresses this somewhat, by demonstrating that only limited variation in workplace productivity is seen between university teaching and DGHs, or between urban and rural hospitals. In addition, at each training site responsible for delivering the core surgical training curriculum, minimum JCST and deanery requirements were met by the majority of trainees, irrespective of placement length, suggesting high‐quality training throughout the region. Therefore, it is argued that no disadvantage would result from longer placements in these respective settings, where a single educational supervisor can be complemented by the presence of several clinical supervisors, responsible for delivering clinical skill acquisition. This is particularly true at CST level, where the objective is acquisition of basic surgical skills and further development of skills relating to diagnostics and patient management. However, caution should be taken when appointing individuals to the role of educational supervisor in this setting. Although there are clear benefits, there is potential that a less able trainer, or disengaged educational supervisor, could inhibit rather than promote a trainee's development. To reap the benefits of longer training rotations, plans to professionalize the role of trainers will be key.

Finally, CSTs have been found to be vulnerable to burnout, with 59 per cent reporting burnout in at least one domain on the Maslach Burnout Inventory for healthcare workers^24^. This is likely due to a combination of factors, including stringent curriculum and training goals to be achieved in only a 2‐year period, examination pressures, and the prospect of application for higher surgical training. Moving between hospital sites undoubtedly adds to the pressure on CSTs, and this may be mitigated partially by longer rotations in the same hospital. Longer placements also promote familiarity between trainee and trainer, and a greater understanding of a trainee's needs and competency results. This leads theoretically to more appropriate and individualized training, delivering operative competency progression, increased time to complete academic pursuits, and increased willingness to complete WBAs.

This study represents a complete data set comprising 344 rotations over a 3‐year study period. The study has statistical power, and the findings comprise the first evidence supporting the move towards longer rotations and greater continuity in producing well trained surgeons. Limitations include that the data represent a single deanery experience and that a proportion of the data gathered were largely dependent on the accuracy with which trainees had recorded academic activity within their ISCP profiles. Longer rotations were typically undertaken in the second CST year, and this seniority factor may confound the better workplace productivity associated with longer rotations. Yet, there was still an incremental increase in WBAs, operative numbers and academic productivity identified between 6‐ and 12‐month rotations, despite both being undertaken primarily in the second year of core training.

The impact of training environment continuity should not be underestimated. The findings of this study provide strong objective evidence to support longer rotations in terms of global trainee outcome‐based competencies. Workforce planning allied to training programme reconfiguration as part of IST in the UK should consider these findings in rotational planning, and it is suggested that the core surgical training programme structure be two 6‐month rotations in year one, followed by a themed specialty 12‐month rotation in year two, rotating through a maximum of two hospitals.

## Supporting information


**Table S1** Comparison of workplace‐based assessments, operative experience and academic output related to specialty theme of training
