# Supplementary material for: Surgical training rotation design: effects of hospital type, rotation theme and duration
Source: BJS Open. 2020 Jul 24;4(5):970–6. doi: 10.1002/bjs5.50326 (PMC7528516; doi:10.1002/bjs5.50326)
Supplement: Supplementary file 1 — Table S1 Comparison of workplace‐based assessments, operative experience and academic output related to specialty theme of training [file BJS5-4-970-s001.docx]

**BJS5_50326**

**Surgical training rotation design: effects of hospital type, rotation theme and duration**

**D. B. T. Robinson, L. Hopkins, O. P. James, C. Brown, A. G. M. T. Powell, S. Hemington-Gorse, T. Abdelrahman, W. G. Lewis and R. J. Egan**

**Table S1** Comparison of workplace-based assessments, operative experience and academic output related to specialty theme of training

|  | **Speciality** | | | | | | |  |
| --- | --- | --- | --- | --- | --- | --- | --- | --- |
|  | **GS**  **(n=138)** | **T&O**  **(n=104)** | **ENT**  **(n=34)** | **Urology (n=14)** | **OMFS**  **(n=13)** | **Plastics (n=33)** | **Neurosurgery (n=8)** | **p-value** |
| **CBD+CEX** | 36 (0-120) | 36 (3-99) | 39 (0-87) | 38 (3-76) | 33 (4-48) | 42 (12-90) | 41 (21-75) | 0.778 |
| **Consultant CBD+CEX** | 30 (0-105) | 30 (0-99) | 30 (0-57) | 32 (3-74) | 30 (4-41) | 33 (6-90) | 30 (15-66) | 0.899 |
| **DOPS+PBA** | 32 (0-150) | 27 (3-96) | 32 (0-135) | 26 (6-69) | 24 (3-63) | 36 (0-66) | 27 (18-84) | 0.283 |
| **Consultant DOPS+PBA** | 23 (0-117) | 21 (0-87) | 18 (0-63) | 19 (3-58) | 18 (2-48) | 21 (0-63) | 20 (0-78) | 0.658 |
| **Total WBA** | 70 (0-261) | 69 (10-156) | 80 (6-198) | 61 (24-118) | 66 (7-111) | 72 (27-156) | 72 (39-153) | 0.529 |
| **Total consultant WBA** | 51 (0-189) | 53 (10-144) | 48 (6-90) | 50 (15-110) | 45 (6-84) | 57 (18-153) | 51 (18-144) | 0.959 |
| **Total operative cases** | 249 (54-552) | 249 (93-477) | 273 (117-522) | 220 (134-492) | 324 (132-582) | 270 (96-737) | 225 (69-393) | 0.354 |
| **STS+ operative cases** | 87 (3-357) | 72 (6-291) | 99 (12-333) | 129 (39-394) | 147 (54-263) | 108 (21-366) | 66 (30-138) | **0.009** |
| **Audit** | 0 (0-9) | 0 (0-9) | 0 (0-9) | 0 (0-4) | 0 (0-3) | 0 (0-6) | 0 (0-0) | 0.719 |
| **Presentations** | 0 (0-14) | 0 (0-9) | 0 (0-12) | 0 (0-3) | 0 (0-12) | 0 (0-9) | 0 (0-6) | 0.552 |
| **Publications** | 0 (0-3) | 0 (0-3) | 0 (0-3) | 0 (0-0) | 0 (0-0) | 0 (0-6) | 0 (0-3) | 0.417 |

Results are based on Kruskul-Wallis analysis and are reported as medians with ranges in parentheses. p-value in bold indicates significance. CBD – Case-based discussion; CEX - clinical evaluation exercises; DOPS – direct observation of procedural skills; PBA – Procedure based assessment; STS+ - Supervised trainer scrubbed or greater; GS – general surgery, T&O – trauma and orthopaedics, ENT – ear, nose and throat surgery, OMFS – oral and maxillofacial surgery
